# Pleural fluid osteopontin, vascular endothelial growth factor, and urokinase-type plasminogen activator levels as predictors of pleurodesis outcome and prognosticators in patients with malignant pleural effusion: a prospective cohort study

**DOI:** 10.1186/s12885-016-2529-1

**Published:** 2016-07-13

**Authors:** Li-Han Hsu, Pei-Chi Hsu, Tien-Ling Liao, An-Chen Feng, Nei-Min Chu, Shu-Huei Kao

**Affiliations:** Ph.D. for Medical Biotechnology Program, College of Medical Science and Technology, Taipei Medical University, Taipei, Taiwan; Department of Medicine, National Yang-Ming University Medical School, Taipei, Taiwan; Division of Pulmonary and Critical Care Medicine, Sun Yat-Sen Cancer Center, Taipei, Taiwan; School of Medical Laboratory Science and Biotechnology, College of Medical Science and Technology, Taipei Medical University, 250 Wu-Hsing Street, Taipei, 110 Taiwan; Department of Research, Sun Yat-Sen Cancer Center, Taipei, Taiwan; Department of Medical Oncology, Sun Yat-Sen Cancer Center, Taipei, Taiwan

**Keywords:** Malignant pleural effusion, Osteopontin, Pleurodesis, Survival, Urokinase-type plasminogen activator, Vascular endothelial growth factor

## Abstract

**Background:**

Rapidly growing cancer cells secrete growth-promoting polypeptides and have increased proteolytic activity, contributing to tumor progression and metastasis. Their presentation in malignant pleural effusion (MPE) and their predictive value for the outcome of pleurodesis and survival were studied.

**Methods:**

Between February 2011 and March 2012, MPE samples were prospectively collected from 61 patients. Twenty-five patients with non-malignant pleural effusion in the same period were included as controls. Pleural fluid osteopontin (OPN), vascular endothelial growth factor (VEGF), and urokinase-type plasminogen activator (uPA) concentrations were measured.

**Results:**

Patients with MPE had higher pleural fluid OPN, VEGF, and uPA concentrations than those with non-malignant pleural effusion, but only differences in VEGF were statistically significant (*p* = 0.045). Patients with distant metastases had significantly elevated pleural fluid VEGF concentrations than those without (*p* = 0.004). Pleural fluid OPN, VEGF, and uPA concentrations were positively correlated in most patients. However, there was no significant difference in pleural fluid OPN, VEGF, and uPA concentrations between patients with successful pleurodesis and those without. There was also no significant difference in cancer-specific survival between sub-groups with higher and lower pleural fluid OPN, VEGF, or uPA concentrations. Patients with successful pleurodesis had significantly longer cancer-specific survival than those without (*p* = 0.015).

**Conclusions:**

Pleural fluid OPN, VEGF, and uPA concentrations are elevated in MPE but are not satisfactory predictors of pleurodesis outcome or survival. Patients with higher pleural fluid VEGF concentration have higher risk of distant metastasis. Evaluating the benefits of therapy targeting the VEGF pathway in these patients warrants further studies.

## Background

Malignant pleural effusions (MPE) frequently cause respiratory compromise in cancer patients. The characteristics of malignant pleural fluid vary widely, from free-flowing to fibrinous and from sero-sanguinous to bloody. Drainage followed by instillation of sclerosing agents is often used to prevent pleural fluid accumulation and to improve the patient’s quality of life [1–3]. However, the efficacy of this treatment varies and its effect on cancer survival is uncertain. Rapidly growing cancer cells frequently secrete growth-promoting polypeptides and have increased proteolytic activity. Several studies indicate that growth factors have an ability to modulate the expression of proteolytic enzymes [4]. Osteopontin (OPN), a small integrin-binding ligand N-linked glycoprotein, is emerging as an important player in regulating cell signaling that controls tumor progression and metastasis [5, 6]. Vascular endothelial growth factor (VEGF) can increase vascular permeability and the proliferation and migration of endothelial cells. Both OPN and VEGF are involved in the production of urokinase-type plasminogen activator (uPA) and the formation of MPE [7–10].

The balance of plasminogen activator and plasminogen activator inhibitor (PAI) determines the pro-coagulant and fibrinolytic activities within the pleural space [11, 12]. Increased intra-pleural fibrinolysis will lead to failure of pleurodesis [13, 14]. On the other hand, an over-expression of pro-coagulant activities precipitates fibrin deposition and results in loculated malignant pleural effusions or trapped lungs [15–19]. The fibrin-generation process is reported to prohibit tumor cell invasion and metastasis [20–24].

In this study, pleural fluid OPN, VEGF, and uPA concentrations from patients with MPE on its initial presence were measured and their correlations determined. Under the hypothesis that higher pleural fluid OPN, VEGF, and uPA concentrations could be a predictor of pleurodesis failure or poor prognosis in patients with MPE, their associations with pleurodesis outcome and cancer-specific survival were investigated.

## Methods

### Patients

This prospective study was approved by the Institutional Review Board of the Sun Yat-Sen Cancer Center and by the hospital’s Ethics Committee. It was conducted in accordance with the ethical principles of the Declaration of Helsinki and guidelines on good clinical practice. All of the patients provided written informed consent.

Sixty-one consecutive patients who were symptomatic from MPE on its initial presence were prospectively recruited between February 2011 and March 2012. Their mean age was 57 years and 45 were women (Table 1). Of the 61 patients, 32 had lung cancer, 19 had breast cancer, and four had ovarian cancer, while transitional cell carcinoma, colon cancer, melanoma, pancreatic cancer, lymphoma, and unknown primary adenocarcinoma had one patient each. Forty-four patients had distant metastasis other than MPE (20 with lung cancer, 16 with breast cancer, 3 with ovarian cancer, and one each with transitional cell carcinoma, colon cancer, melanoma, pancreatic cancer, and unknown primary adenocarcinoma).

**Table 1:** Characteristics of patients with malignant pleural effusion

	All	Lung cancer	Breast cancer	Others
Number	61	32	19	10
Age^a^	57.0±11.8	59.9±12.9	53.6±11.1	55.0±8.5
Sex				
Male	16	12	0	4
Female	45	20	19	6
Pre-menopausal	16	7	7	2
Post-menopausal	29	13	12	4
Distant metastasis				
Yes	44	20	16	8
No	17	12	3	2

^a^Data are presented as mean ± standard deviation

Median survival was 198 days after the presence of MPE was noted (128 days in lung cancer and 222 days in breast cancer). Pleural fluid samples were obtained by standard thoracentesis using size 8–14 Fr self-retaining intra-pleural catheter (Pigtail drainage tube; Create Medic, Yokohama, Japan). The MPE was confirmed by cell block cytology or closed pleural biopsy. The pleural fluid samples were immediately centrifuged to remove cells and debris, and then stored at -80 °C for analysis.

Twenty-six patients had good ipsilateral lung re-expansion when the intra-pleural catheter drainage was stopped. They were eligible for chemical pleurodesis with minocycline (Lederle Parenterals, Carolina, Puerto Rico). Follow-up chest radiographs were obtained at 1, 3, and 6 months after pleurodesis and repeated as needed. Fifteen (six with breast cancer, six with lung cancer, and one each with ovarian cancer, lymphoma, and unknown primary adenocarcinoma) achieved complete or partial success. Eleven (six with breast cancer and five with lung cancer) had failed pleurodesis according to the definitions proposed by the American Thoracic Society and the European Respiratory Society Consensus Statement [1]. The remaining patients who were not suitable for pleurodesis because of loculated pleural effusion or trapped lung were also followed.

Twenty-five patients (15 women; mean age, 64 years) with non-malignant pleural effusion in the same period were included as controls. Their etiologies were indeterminate lymphocyte predominant exudate (*n* = 13), para-pneumonia (*n* = 3), heart failure (*n* = 3), surgery or radiotherapy (*n* = 3), transudates secondary to liver cirrhosis or metastasis (*n* = 2) and pulmonary embolism (*n* = 1). All pleural fluid samples were examined with cell block and confirmed negative for malignancy.

### Measurement of pleural fluid OPN, VEGF, uPA, and PAI-1 concentrations

The concentrations of OPN, VEGF, uPA, and PAI-1 in the supernatants of the pleural fluid samples were measured by commercially available enzyme-linked immuno-sorbent assay kits: OPN (TiterZyme® EIA Human Osteopontin Enzyme Immunometric Assay Kit; Assay Designs, Ann Arbor, MI, USA), VEGF (Human VEGF Immunoassay kit; Invitrogen, Camarillo, CA, USA), uPA (u-PA Activity Assay Kit; Chemicon, Temecula, CA, USA), and PAI-1 (PAI Activity Assay Kit; Chemicon). The concentrations of OPN and uPA were expressed as ng/mL, while the concentrations of VEGF and PAI-1 were expressed as pg/mL.

Aside from numerical data, the OPN, VEGF, uPA, and PAI-1 concentrations were also coded as high or low using median cut-offs. To relate uPA and PAI-1 with clinical outcome, a binary variable, uPA/PAI-1 was evaluated, since previous studies had shown that the combination of both markers provided better risk-group discrimination than either one alone [24].

Pleural fluid OPN, VEGF, uPA, and PAI-1 concentrations were compared between patients with MPE and those with non-malignant pleural effusion and between sub-groups of MPE patients divided by sex, underlying malignancy, and presence of distant metastasis. Pre-menopausal status was associated with more advanced disease and a shorter survival among never-smoking female patients with lung adenocarcinoma, implying an estrogen cancer-promoting effect [25], comparison was also made between the pre- and post-menopausal women. Linear regression analyses were performed to measure correlations among pleural fluid OPN, VEGF, uPA, and PAI-1 concentrations in MPE.

### Investigation of the association between pleural fluid OPN, VEGF, uPA, and PAI-1 concentrations and pleurodesis outcome and survival

Pleural fluid OPN, VEGF, uPA, and PAI-1 concentrations were compared between patients with successful pleurodesis and those without. Kaplan-Meier plots and log-rank tests were used to assess the association between pleural fluid OPN, VEGF, uPA, and PAI-1 concentrations and cancer-specific survival. Log-rank test was also used to compare cancer-specific survival between patients with and those without successful pleurodesis.

### Statistical analysis

Descriptive statistics of mean, median, standard deviation, and frequency were used to process the demographic and laboratory data. Categorical variables were compared using Chi-square test, while continuous variables were compared using independent *t* test. Statistical significance was set at *p* < 0.05. All analyses were performed using the statistical software package SAS, version 9.4 (SAS Institute; Cary, NC, USA).

## Results

### Patients with MPE had significantly higher pleural fluid VEGF concentrations, especially in those with distant metastases

Patient with MPE had higher pleural fluid OPN (mean, 826.85 ± 161.45 vs. 655.88 ± 84.83 ng/mL; *p* = 0.578), VEGF (mean, 3720.72 ± 747.19 vs. 2036.15 ± 317.58 pg/mL; *p* = 0.045), and uPA (mean, 52.25 ± 14.60 vs. 28.03 ± 2.96 ng/mL; *p* = 0.274) concentrations than those with non-malignant pleural effusion. However, only the difference in VEGF concentration reached statistical significance. There was no significant difference in pleural fluid OPN, VEGF, uPA, and PAI-1 concentrations between different sub-groups except for those with distant metastases in addition to MPE. They had significantly higher pleural fluid VEGF concentrations than those without distant metastases (mean, 4624.28 ± 990.75 vs. 1382.08 ± 458.78 pg/mL; *p* = 0.004) (Table 2).

**Table 2:** Pleural fluid OPN, VEGF, uPA, and PAI-1 concentrations in patients with malignant pleural effusion

	OPN (ng/mL)	*p* value	VEGF(pg/mL)	*p* value	uPA(ng/mL)	*p* value	PAI-1(pg/mL)	*p* value
All	826.85 ± 161.45		3720.72 ± 747.19		52.25 ± 14.60		1228.42 ± 69.74	
Sex		0.666		0.649		0.079		0.788
female	784.84 ± 172.59		3515.24 ± 897.44		61.25 ± 19.40		1236.17 ± 87.96	
male	945.01 ± 387.95		4298.61 ± 1356.14		25.84 ± 3.53		1203.24 ± 83.89	
Female		0.168		0.905		0.549		0.854
Pre-menopausal	1147.57 ± 381.79		3375.61 ± 1146.41		48.21 ± 17.76		1215.35 ± 115.62	
Post-menopausal	564.60 ± 144.27		3600.02 ± 1279.00		68.70 ± 28.92		1249.18 ± 125.16	
Pathology		0.497		0.938		0.615		0.788
lung cancer	990.10 ± 273.93		3270.75 ± 866.73		47.47 ± 13.33		1243.12 ± 94.15	
breast cancer	752.21 ± 213.94		3163.96 ± 1015.91		37.52 ± 12.82		1203.66 ± 102.48	
Distant metastasis		0.720		0.004		0.485		0.855
Yes	790.42 ± 186.64		4624.28 ± 990.75		57.50 ± 19.34		1236.77 ± 88.35	
No	921.14 ± 128.15		1382.08 ± 458.78		39.27 ± 17.25		1208.38 ± 110.42	

Data are presented as mean ± standard deviation

### Positive correlations among pleural fluid OPN, VEGF, and uPA concentrations in patients with MPE

Pleural fluid OPN, VEGF, and uPA concentrations positively correlated with each other (Table 3). The correlation coefficient between pleural fluid OPN and VEGF concentrations was stronger than that between VEGF and uPA, and that between OPN and uPA. Pleural fluid uPA and PAI-1 concentrations were negatively correlated.

**Table 3:** Relationships between pleural fluid OPN, VEGF, uPA, and PAI-1 concentrations in patients with malignant pleural effusion

	All	Male	Female	Female		Lung cancer	Breast cancer	Distant metastasis	
				Pre-menopausal	Post-menopausal			Yes	No
OPN & VEGF									
*r*	0.466	0.750	0.279	−0.105	0.824	0.493	0.709	0.503	0.655
*p* value	<0.001	0.032	0.005	0.088	0.023	0.010	0.029	0.001	0.462
VEGF & uPA									
*r*	0.244	0.894	0.282	0.717	−0.047	0.360	−0.034	0.344	−0.108
*p* value	< 0.001	0.006	< 0.001	0.010	0.002	< 0.001	0.005	< 0.001	0.010
OPN & uPA									
*r*	0.068	0.642	0.054	0.074	−0.009	0.081	0.008	0.145	−0.104
*p* value	< 0.001	0.017	< 0.001	0.011	< 0.001	< 0.001	0.001	< 0.001	0.010
uPA & PAI-1									
*r*	−0.682	−0.612	−0.689	−0.808	−0.618	−0.679	−0.717	−0.652	−0.831
*p* value	< 0.001	< 0.001	< 0.001	< 0.001	< 0.001	< 0.001	< 0.001	< 0.001	< 0.001

*r*, correlation coefficient

In sub-group analysis, the correlation coefficient between pleural fluid OPN and VEGF concentrations was stronger in males, post-menopausal females, lung cancer patients, breast cancer patients, and patients with distant metastases. The correlation coefficient between pleural fluid VEGF and uPA concentrations was stronger in males and pre-menopausal females. The correlation coefficient between pleural fluid OPN and uPA concentration was stronger in males. Pleural fluid uPA and PAI-1 concentrations were negatively correlated across different sub-groups.

### There were no associations of pleural fluid OPN, VEGF, uPA and PAI-1 concentrations with pleurodesis outcome and survival

There was no significant difference in pleural fluid OPN (mean, 809.53 ± 287.72 vs. 361.54 ± 71.80 ng/mL; *p* = 0.151), VEGF (mean, 5610.94 ± 2040.61 vs. 3564.96 ± 1044.12 pg/mL; *p* = 0.383), uPA (mean, 99.04 ± 53.88 vs. 25.80 ± 3.22 ng/mL; *p* = 0.198), and PAI-1 (mean, 1099.87 ± 130.85 vs. 1306.31 ± 106.46 pg/mL; *p* = 0.240) concentrations or uPA/PAI-1 ratio (*p* = 0.336) between patients with and those without successful pleurodesis.

When stratified at the median, there was no significant difference in cancer-specific survival between patients with higher and lower pleural fluid OPN (median, 128 vs. 138 days; *p* = 0.773), VEGF (median, 127 vs. 147 days; *p* = 0.531), uPA (median, 128 vs. 145 days; *p* = 0.232), and PAI-1 (median, 178 vs. 108 days; *p* = 0.831) concentrations or uPA/PAI-1 ratio (median, 128 vs. 138 days; *p* = 0.710). Since patients with lung cancer and MPE had shorter survival than those with breast cancer, survival analyses were also separately done on the lung cancer and breast cancer sub-group to determine the effect of tumor heterogeneity.

In the lung cancer sub-group, patients with higher pleural OPN had shorter cancer-specific survival (median, 113 vs. 146 days; *p* = 0.026). There was no significant difference in cancer-specific survival between patients with higher and lower pleural fluid VEGF, uPA, and PAI-1 concentrations or uPA/PAI-1 ratio. In the breast cancer sub-group, there was no significant difference in cancer-specific survival between patients with higher and lower pleural fluid OPN, VEGF, uPA, and PAI-1 concentrations or uPA/PAI-1 ratio.

**Fig. 1:**
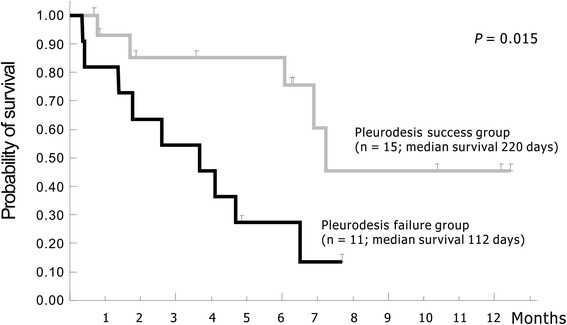
Patients with successful pleurodesis had significantly longer cancer-specific survival than those without

Patients with successful pleurodesis had significantly longer cancer-specific survival than those without (median, 220 vs. 112 days; *p* = 0.015) (Fig. 1).

## Discussion

Recent research has elucidated the pivotal role of OPN in regulating cell signaling that controls tumor progression and metastasis. Enhanced OPN expression has been noted in the plasma of advanced lung cancer patients and associated with MPE [26–29]. Moreover, OPN has been speculated to be involved in the formation of MPE [30]. On the other hand, VEGF is a mitogen for endothelial cells involved in angiogenesis and is a potent inducer of vascular permeability. It has also been reported as a key mediator in pleural fluid formation [9]. Together, OPN has been reported to up-regulate VEGF through the Akt activation and the induction of HIF-1α expression [31]. In the present study, the positively correlated pleural fluid OPN and VEGF concentrations across different sub-groups support the assumption that OPN can induce the release of VEGF and promote MPE formation [30, 32–34].

The plasminogen activator system consists of plasminogen activators (urokinase and tissue-type plasminogen activators), their receptors, and their inhibitors. Plasminogen activators split plasminogen to plasmin, which can then break down extracellular matrix components. Thus, OPN-induced cancer cell migration and invasion requires uPA [35–37], while VEGF can induce pro-uPA activation on the surface of endothelial cells. The angiogenic response of endothelial cells initiated by VEGF is accompanied by the assembly of cell surface-bound proteolytic machinery as a prerequisite for focal invasion [38, 39]. Stepwise analysis also identifies uPA as an independent factor correlated with micro-vessel density [40].

The positively correlated pleural fluid OPN and uPA, and VEGF and uPA concentrations in patients with MPE provide clinical evidences of prior laboratory findings. Additional experiments are needed to clarify the sequence of action of OPN, VEGF, and uPA in the formation of MPE, which may include quantification of pleural fluid mRNA by quantitative real-time PCR, immuno-histochemical staining of freshly isolated pleural cells, measurement of their production by cultured cells from pleural fluids, in situ mRNA hybridization, and zymography to evaluate the spatial and temporal expression [41–44].

Tumor tissue expression and plasma concentration of OPN and VEGF have been correlated with advanced stage and poor survival in most common malignancies, although a small number of studies have also shown no correlation [45]. In patients with lung cancer and MPE, Zhang et al. reported that OPN in pleural effusion was an independent prognostic factor [46]. Kotyza et al. and Hooper et al. demonstrated a significant association between increased pleural fluid VEGF and reduced survival in malignant pleural effusion [47, 48]. Except for OPN in the lung cancer sub-group, the present study did not find any survival difference between sub-groups with higher and those with lower pleural fluid OPN, VEGF, or uPA concentrations in the others. The conflicting results may be attributed to the heterogeneity of the underlying malignancies, with different expected survival rates, small number of each cancer sub-group, and different specimens tested.

Survival analysis was also different across studies. While some employed the log-rank test applied to Kaplan Meier curves above and below the cut-off value of each parameter, others used Spearman’s rank correlation. Further validation of the pleural fluid processing, measurement, and unification of the statistical methods are necessary. The introduction of chemotherapy or targeted therapy before the presence of MPE also affected the survival analysis compared to chemotherapy- or targeted therapy-naïve patients with MPE as one of the first manifestations at diagnosis.

However, compared to OPN or uPA, VEGF is significantly higher in patients with MPE than in the controls, especially in patients with distant metastases in addition to MPE. The potential relevance of pleural fluid VEGF concentration in selecting patients undergoing adjuvant treatment targeting the VEGF pathway is a particularly interesting area for future research.

Although the uPA concentration is elevated in the MPE, the more substantially elevated concentrations of its inhibitors, namely PAI-1 and PAI-2, likely result in depressed fibrinolytic activity and favor the deposition and maintenance of intra-pleural fibrin. Pedersen et al. proposed that PAI-1 served to protect cancer tissue against the proteolytic degradation that the tumor imposed upon the surrounding normal tissue [49, 50]. In contrast, breast cancer patients with high uPA/PAI-1 ratio in tumor tissue extracts are reported to have increased relapse risk [23, 24]. The failure to counterbalance the exuberant production of uPA characteristic of tumor cells by the timely production of sufficient PAI-1 by host cells may contribute to neoplastic proliferation and formation of metastasis. The latter is more consistent with the hypothesis of the present study. However, there is no association of increased pleural fluid uPA concentrations or uPA/PAI-1 ratio with lower success rate of pleurodesis or shorter survival. Such contradictory results may be attributed to the case number of this study, which may be too small to discern differences and different specimens tested.

Pleurodesis is traditionally regarded as symptomatic treatment, with rare mention in literature of its benefit on survival. Hirata et al. reported that patients with breast cancer and MPE receiving systemic therapy following the initial talc pleurodesis had longer survival than those receiving systemic therapy alone [51]. Recently, Korsic et al. reported that patients who underwent successful talc pleurodesis had significantly longer survival than patients in the control group treated with serial thoracentesis, especially in the groups with breast cancer and better performance status [52]. Antony et al. demonstrated a strong apoptotic effect of talc on cells of malignant mesothelioma *in vitro* [53]. They also found that pleural mesothelial cells released great amounts of endostatin after pleurodesis [54].

Talc alters angiogenic balance in the pleural space from a biologically active and angiogenic environment to an angiostatic milieu. However, the above-mentioned survival benefit does not seem to be limited to talc. Patients with successful minocycline pleurodesis in this study also have significantly longer survival than those with failed pleurodesis (Fig. 1). The fibrin-generation potential of patients following installation of sclerosing agent is decisive for pleurodesis outcome and survival. Successfully induced pleural fibrosis is supposed to prohibit tumor invasion and metastasis. Recruiting more patients with MPE in the future may help to confirm these findings.

To date, this is the first study to prospectively evaluate the presentations and relationship among pleural fluid OPN, VEGF, and uPA concentrations in patients with MPE and examine their association with pleurodesis outcome and survival. There are several limitations that need to be overcome, including the small sample size and heterogeneity of underlying malignancies.

## Conclusions

Although elevated in malignant pleural effusion, pleural fluid OPN, VEGF, and uPA concentrations are neither satisfactory predictors of pleurodesis outcome or prognosticators from the current study. However, patients with higher pleural fluid VEGF concentration have a higher risk of distant metastasis. Further study is required to evaluate the benefit of this sub-group from therapy targeting the VEGF pathway.

## Abbreviations

MPE, malignant pleural effusion; OPN, Osteopontin; PAI-1, plasminogen activator inhibitor-1; uPA, urokinase-type plasminogen activator; VEGF, vascular endothelial growth factor.
